# Heterotrophic ammonia oxidation by *Alcaligenes* balances ROS generation and terminal electron transport

**DOI:** 10.1002/mlf2.70035

**Published:** 2025-09-29

**Authors:** Runhua Wang, Xiaokang Wang, Yue Zhao, Xize Zhao, Tong Wu, Yulin Wang, Ruofei Li, Jun Yao, Chengying Jiang, Ji‐Guo Qiu, De‐Feng Li, Shuang‐Jiang Liu

**Affiliations:** ^1^ State Key Laboratory of Microbial Diversity and Innovative Utilization, Institute of Microbiology Chinese Academy of Sciences Beijing China; ^2^ University of Chinese Academy of Sciences Beijing China; ^3^ China University of Geosciences Beijing China; ^4^ State Key Laboratory of Microbial Technology Shandong University Qingdao China; ^5^ Key Laboratory of Agricultural and Environmental Microbiology, Ministry of Agriculture and Rural Affairs, College of Life Sciences Nanjing Agricultural University Nanjing China

**Keywords:** *Alcaligenes*, Dirammox, heterotrophic ammonia oxidation

## Abstract

Heterotrophic nitrifiers are bacteria that aerobically oxidize ammonia in the presence of organic carbon sources, which differs from autotrophic nitrifiers that extract energy from ammonia oxidation for cell metabolism and growth. The physiological significance of heterotrophic ammonia oxidation remains unclear, even though this process has been known for decades. Here, we demonstrate that direct ammonia oxidation (Dirammox)—a heterotrophic ammonia oxidation process with dinitrogen (N_2_) as the primary product—is associated with both redox balance and the electron transport chain in *Alcaligenes faecalis*. Genetic and proteomic studies indicated that disruption of Dirammox genes (*dnfA/dnfB/dnfC*) induces a transient redox imbalance and perturbation in energy metabolism, further resulting in delayed growth. In addition, we found via biochemical and physiological studies that endogenous reactive oxygen species (ROS) enhance redox fluxes to ammonia oxidation, and the genetic disruption of cytochrome *c* peroxidase results in an increased flux of electrons to ammonia oxidation, producing N_2_ and N_2_O. These unexpected findings provide a more thorough understanding of both the Dirammox process and the physiology of heterotrophic ammonia oxidation.

## Impact statement

Although heterotrophic ammonia‐oxidizing bacteria have been identified for decades, their biochemical and physiological roles remain largely unknown. Our results unveiled the association between ammonia oxidation (Dirammox), redox balance, and the electron transport chain, revealing the essential physiological role of ammonia oxidation in heterotrophic ammonia oxidizers and nitrifiers. This study provides a theoretical foundation for enhancing the ammonia oxidation activity of the Dirammox and potentially guiding the construction of highly efficient nitrogen‐removing microorganisms.

## INTRODUCTION

Atmospheric dinitrogen gas (N_2_) is the largest inventory of nitrogen. Ammonia is the most reduced and reactive form of nitrogen for biological processes, and the microbial transformation and oxidation of ammonia are essential and key processes for transforming reactive nitrogen compounds back to N_2_ ^1^. Chemolithoautotrophic ammonia‐oxidizing bacteria (AOB) and ammonia‐oxidizing archaea (AOA) utilize ammonia as an energy source^2^, ^3^. Both AOB and AOA initially oxidize ammonia to hydroxylamine with ammonia monooxygenase (AMO) and then to nitrite via octahaem hydroxylamine oxidoreductases (HAO)^4^, ^5^. Aside from chemolithotrophic nitrifiers, heterotrophic bacteria/nitrifiers have been known for decades to perform ammonia oxidation and nitrification^6^, ^7^, ^8^, ^9^. Chemolithotrophic nitrifiers extract energy from ammonia oxidation for cell metabolism and growth, whereas heterotrophic nitrifiers obtain energy for growth and cellular activity via oxidation of organic compounds. Although this difference between chemolithotrophic nitrifiers and heterotrophic nitrifiers is known, the physiological relevance of why heterotrophic nitrifiers oxidize ammonia is unknown.

Understanding the biochemical and genetic mechanisms underlying heterotrophic nitrification may help researchers better understand this process. The heterotrophic nitrifiers *Pseudomonas* sp.^6^, ^7^, *Arthrobacter* sp.^8^, ^9^, and *Paracoccus* sp.^10^ produce nitrite (NO_2_^–^) and nitrate (NO_3_^–^) from ammonia oxidation. The genus *Alcaligenes*, a group of bacteria widely distributed in the soil, as well as in both natural and artificial aquatic environments, oxidizes ammonia to NH_2_OH, NO, N_2_O, and N_2_, in addition to NO_2_^–^ and NO_3_^–^ in Refs.^11^, ^12^, ^13^, ^14^. The significance of NH_2_OH production went unnoticed until the discovery of the heterotrophic and nitrifying *Alcaligenes ammonioxydans* strain HO‐1. NH_2_OH molecules react with aldehyde, resulting in oxime formation in cells, and oxime is further oxidized to NO_2_^–^ by a pyruvic oxime dioxygenase (POD)^15^. *A. ammonioxydans* strain HO‐1 transiently accumulates large amounts of NH_2_OH from ammonia oxidation, which is not dependent on POD, and the accumulated NH_2_OH is subsequently converted into N_2_ in the presence of O_2_ ^16^. This aerobic oxidation of ammonia via a N_2_‐producing process is termed Dirammox (direct ammonia oxidation), which differs from denitrification in that N_2_ is produced from the reduction of NO_3_^–^. It also differs from Comammox in that NH_3_ is oxidized completely to NO_3_^–^, and it differs from Anammox, as that process occurs anaerobically.

Currently, the genetics and enzymology of heterotrophic ammonia oxidation remain largely unknown. A historically putative ammonia monooxygenase gene (GenBank accession number Y14338) was proposed for *Pseudomonas putida* strain DSMZ‐1088‐260^7^, but this gene likely encodes an ammonia oxygenase because the deletion of its homolog *AFA_16745* in *A. faecalis* did not interrupt ammonia oxidation^17^. Recently, the gene cluster *dnfABC*, which was identified and cloned from the HO‐1 genome, enabled recombinant *E. coli* cells to generate ^15^NH_2_OH and ^30^N_2_ from (^15^NH_4_)_2_SO_4_ ^16^. Based on bioinformatic analyses, the translational products of *dnfA*, *dnfB*, and *dnfC* were annotated as a diiron oxygenase, NADH‐dependent reductase, and glutamine amidotransferase, respectively, all of which are essential for the Dirammox process^17^, ^18^. In the presence of molecular O_2_ and NADH, purified DnfA catalyzed the conversion of NH_2_OH into N_2_ in vitro^18^, ^19^. A more recent study reported that NH_2_OH can be transformed into N_2_ via abiotic reactions^20^, which indicated a second route for N_2_ formation via Dirammox. In addition, increasing evidence suggests that ammonia oxidation in *A. faecalis* is initiated by DnfA with the assistance of DnfB via intermediates of glutamine or l‐glutamic acid γ‐hydroxamate that is subsequently hydrolyzed by DnfC to NH_2_OH^18^, ^19^. Since homologs of DnfA/DnfB/DnfC have been identified in several bacterial genera, including *Pseudomonas*, *Delftia*, and *Microvigula* ^19^, ^21^, the Dirammox process might occur widely in heterotrophic bacteria.

Although the biochemistry and genetics of ammonia oxidation are slowly being elucidated, the physiology of heterotrophic nitrification remains unknown. Dirammox has been suggested, and experimentally confirmed, to benefit *A. faecalis* HO‐1 by detoxifying high concentrations of ammonia^18^, ^22^, ^23^. Considering the wide occurrence of heterotrophic nitrifiers, it is reasonable to hypothesize that heterotrophic nitrification plays physiological and fundamental roles. In this study, we aimed to unravel the association of Dirammox with its host physiological processes. By utilizing multiple research tools, we unexpectedly discovered that Dirammox is associated with cellular redox balance and the electron transport chain in *A. faecalis*, suggesting that heterotrophic ammonia oxidation plays an essential role in the growth and metabolism of *A. faecalis* via Dirammox.

## RESULTS

### The Δ*dnfA*/*B*/*C* mutants show delayed growth and respiration heat release

**Figure 1:**
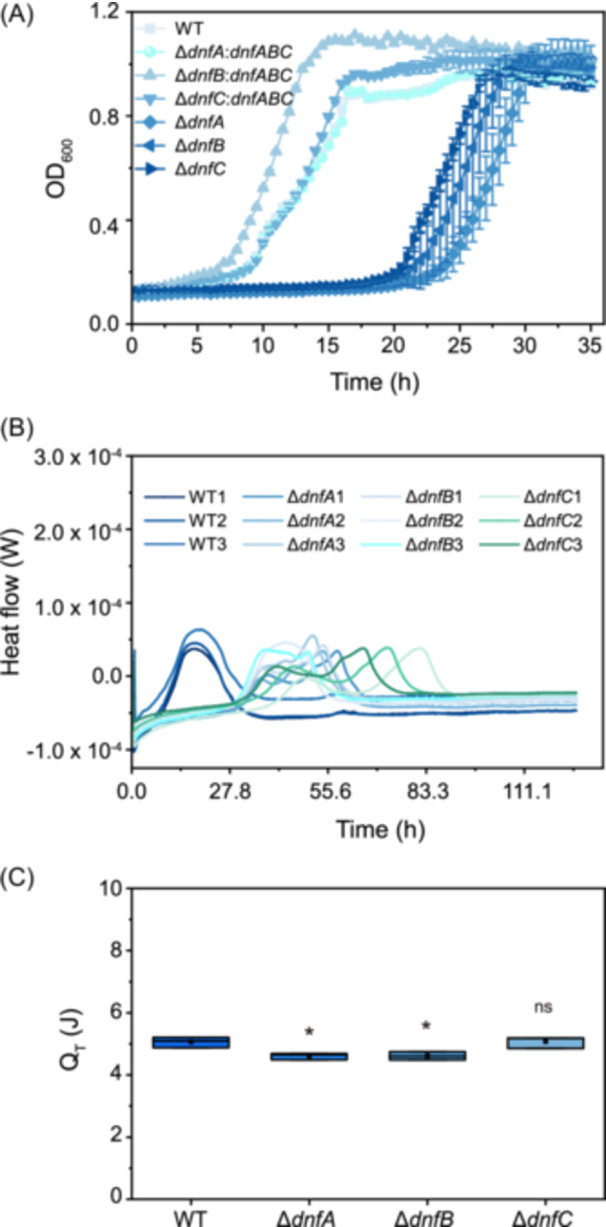
Growth of and release of respiration heat by *Alcaligenes faecalis* wild‐type (WT) and Δ*dnfA/B/C* mutants. (A) Growth curves of WT, Δ*dnfA*, Δ*dnfB*, Δ*dnfC*, Δ*dnfA*:*dnfABC* (Δ*dnfA* strain supplemented with the *dnfABC* cluster), Δ*dnfB*:*dnfABC* (Δ*dnfB* strain supplemented with the *dnfABC* cluster), and Δ*dnfC*:*dnfABC* (Δ*dnfC* strain supplemented with the *dnfABC* cluster) cultured in heterotrophic nitrification medium (HNM). (B) Heat flow curves of the WT and mutant strains cultured in HNM. (C) Respiration heat (*Q*_T_) of the WT and mutant strains. Statistically significant differences were determined using a Student *t*‐test. Significance was based on comparisons to the WT group. ns, no significance; *p < 0.05.

With continuous efforts to understand the process of Dirammox^17^, ^18^, ^19^, ^24^, we did not observe differences in the growth of wild‐type (WT) *A. faecalis* JQ135 and Δ*dnfA/B/C* mutants in Luria–Bertani (LB) broth (Figure S1). However, when the cells grown in LB were transferred to Heterotrophic Nitrification Medium (HNM), the *A. faecalis* Δ*dnfA*/*B*/*C* mutants showed delayed growth compared with the WT. This delayed growth was restored when the mutants were complemented with the *dnfA*, *dnfB*, or *dnfC* genes, demonstrating that these genes play a critical role in growth adaptation in HNM (Figure 1A). Bacterial growth is coupled with the release of respiration heat (*Q*_T_); thus, we further confirmed this observation of delayed growth by monitoring the release of respiration heat by the WT and mutants. We found that the peak of respiration heat release by the mutants was also delayed and was consistent with the growth patterns of the mutants (Figure 1B). We did not observe significant differences in the final cell densities (as indicated with OD_600_) or the total releases of respiratory heat between WT and Δ*dnfC* mutants, although mutants Δ*dnfA* and Δ*dnfB* demonstrated reduced *Q*_T_ compared with the WT (Figure 1A,C).

Considering that auxotrophies for amino acids or vitamins are frequent factors that delay or even stop bacterial growth, we cultivated the Δ*dnfA*/*B*/*C* mutants in HNM supplemented with 20 types of amino acids or a vitamin mix (Figure S2). The results showed that the addition of those nutrients did not restore the growth of the mutants, which indicated that the delayed growth in HNM was not due to auxotrophy.

### The WT and Δ*dnfA*/*B*/*C* mutants have significant differences in proteomic profiles

**Figure 2:**
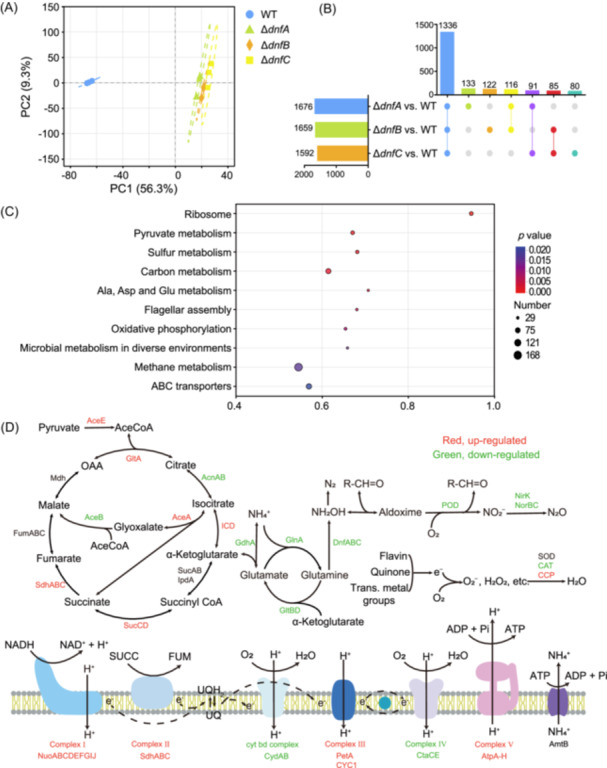
Proteomic profiling of the WT, *ΔdnfA, ΔdnfB, and ΔdnfC* mutant strains cultured in HNM. (A) Principal component analysis (PCA) plot of all samples. Dashed lines show confidence ellipses, representing the distribution range of the group at a 95% confidence level. The boundaries of these confidence ellipses were calculated using Hotelling's *T* ^2^ test. (B) UpSet Venn diagram presenting the differentially expressed proteins (DEPs) and their overlapping relationships. The left horizontal bar chart represents the number of DEPs in each set and the vertical column chart represents the number of shared DEPs among the selected sets. (C) Scatter plots of the KEGG pathways for those DEPs. The dot size represents the number of proteins and the color represents the *p* value range. (D) Schematic representation of the altered metabolic pathways in carbon metabolism, N metabolism, the electron transport chain, and oxidative stress responses.

To determine the mechanism underlying the growth defect in the mutants, we assessed the proteomic profile from the WT and the Δ*dnfA*, Δ*dnfB*, and Δ*dnfC* mutants. We found that the proteomic profiles between the Δ*dnfA*, Δ*dnfB*, and Δ*dnfC* mutants were highly similar, and they were clearly distinguished from the proteomic profile of the WT (Figure 2A), indicating that deletion of Dirammox genes substantially altered the protein expression patterns of *A. faecalis*. Quantitative proteomic analysis showed that there were 1676, 1659, and 1592 differentially expressed proteins (DEPs, defined as changes of folds >1.2 or <0.83 and *p* < 0.05 with Student's *t* test) in the Δ*dnfA*, Δ*dnfB*, and Δ*dnfC* mutants, respectively, compared with WT (Figure 2B). We utilized the Upset Interaction plot to explore the potential interrelationships of DEPs across the WT and mutants, and found that a subset of 1336 DEPs was shared among the three mutants. KEGG pathway annotation and enrichment analysis demonstrated that the overlapping 1336 DEPs belonged to the categories of ribosome, pyruvate metabolism, sulfur metabolism, carbon metabolism, amino acids (alanine, aspartate, and glutamate) metabolism, flagellar assembly, oxidative phosphorylation, methane metabolism, microbial metabolism in diverse environments, and ABC transporters (Figure 2C). Figure S3 further illustrates the proteins involved in Dirammox, oxidative phosphorylation, carbon metabolism, oxidative responses, and ammonia assimilation. We targeted the DEPs distributed within the *dnf* gene cluster and nitrogen metabolism and found that the proteins encoded within the *dnf* cluster were downregulated, except for DnfR, a MocR‐family transcriptional regulator governing the expression of the *dnf* cluster^17^, ^25^. Proteins involved in ammonia assimilation, including glutamine synthetase (GlnA), glutamate synthase (GltBD), and glutamate dehydrogenase (GdhA), were downregulated, indicating that ammonia assimilation was limited in the Δ*dnfA/B/C* mutants.

Interestingly, we observed upregulation of cytochrome *c* peroxidase (CCP), which is a signal for cellular oxidative stress^26^, ^27^. A large amount of DEPs distributed within the TCA cycle and oxidative phosphorylation were upregulated, including pyruvate dehydrogenase (AceE), citrate synthase (GltA), isocitrate dehydrogenase (ICD), succinate‐CoA ligase (SucCD), succinate dehydrogenase (SdhABCD), isocitrate lyase (AceA), complex I (NuoABCDEFGHIJ), complex III (PetA, CYC1), and complex V (AtpA‐H). In summary, the mutants demonstrated altered oxidative stress, reduced N metabolism, and enhanced TCA cycle and energy supply, especially the electron transport chain (Figure 2D).

### The Dirammox process contributes to the consumption of endogenous ROS

**Figure 3:**
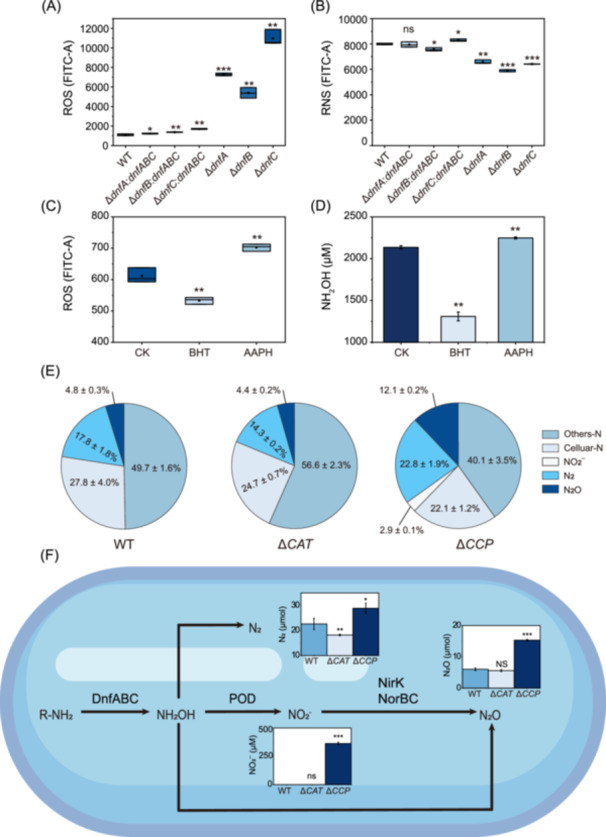
Reactive oxygen species (ROS) is involved in Dirammox and disruption of the *CCP* gene promotes ammonia oxidation to hydroxylamine and dinitrogen (N_2_). (A, B) Detection of total ROS (A) and total reactive nitrogen species (RNS) (B) in the WT, the *ΔdnfA, ΔdnfB, and ΔdnfC* mutants, and their complementary strains. All strains were cultured in HNM. Total ROS and total RNS were detected by fluorescent probe. (C) Total ROS levels in the control (CK), BHT‐, and AAPH‐treated groups. (D) Accumulation of NH_2_OH after a 3‐h incubation with BHT or AAPH. (E) N‐balance experiments of WT, Δ*CAT*, and Δ*CCP*. (F) Silencing of *CCP* enhancing the ammonia oxidation process. All statistically significant differences were determined using a Student *t*‐test. In (A) and (B), significance is based on comparisons to the WT group. In (C) and (D), significance is compared with the CK group. ns, no significance; ***p < 0.001; **p < 0.01; *p < 0.05.

The proteomic profiling showed that the Δ*dnfA*, Δ*dnfB*, and Δ*dnfC* mutants had altered protein profiles for oxidative stress, suggesting a potential correlation between Dirammox and ROS. Thus, we measured the total endogenous ROS and reactive nitrogen species (RNS) levels in WT and the Δ*dnfA*, Δ*dnfB*, and Δ*dnfC* mutants (Figure 3A,B). We found that the total endogenous ROS levels in the Δ*dnfA*, Δ*dnfB*, and Δ*dnfC* mutants were significantly higher than those in WT, while the total endogenous RNS levels in the Δ*dnfA*, Δ*dnfB*, and Δ*dnfC* mutants were lower than those in WT. The complementary strains showed similar levels of total ROS and RNS to those of the WT. We also assessed the individual endogenous O_2_^–^, H_2_O_2,_ and OH^•^ species (Figure S4). In Figure S4A, we observed that the O_2_^–^ levels in the mutants (Δ*dnfA*, Δ*dnfB*, and Δ*dnfC*) increased compared with the WT. However, the O_2_^–^ levels in the mutants were not significantly increased compared with the respective complementary strain (Δ*dnfB:dnfABC* and Δ*dnfC*:*dnfABC*), suggesting that O_2_^–^ is not the primary type of ROS in the mutants. In addition, the endogenous H_2_O_2_ (Figure S4B) and OH^•^ (Figure S4C) levels did not increase in the mutants, suggesting the existence of an unknown ROS molecule. Altogether, the deletion of *dnfABC* resulted in total ROS accumulation and RNS reduction in mutants, although the source of total ROS remained undefined.

Furthermore, we were curious if the ROS accumulation influenced ammonia oxidation. To answer this question, the WT strain was incubated with 500 μM butylated hydroxytoluene (BHT, inhibitor of ROS generation) or 100 μM 2,2′‐azodiisobutyramidine dihydrochloride (AAPH, inducer of ROS generation)^28^, ^29^, and then the level of NH_2_OH accumulation was measured after 3 h incubation. The results showed that the BHT inhibitor treatment group had reduced ROS levels and produced less NH_2_OH, while the AAPH inducer treatment group showed increased ROS levels and produced higher NH_2_OH levels compared with the controls (Figure 3C,D). These observations suggested that endogenous ROS might reinforce the process of Dirammox. Considering that Δ*dnfA*, Δ*dnfB*, and Δ*dnfC* mutants had higher levels of ROS compared with the WT, it was reasonable to deduce that the Dirammox process consumed ROS during ammonia oxidation. In addition, the accumulation of endogenous ROS is harmful to cell growth, and the delayed growth of the Δ*dnfA*, Δ*dnfB*, and Δ*dnfC* mutants might be attributed to ROS accumulation.

CCP and CAT are enzymes involved in ROS scavenging^26^, ^27^. Since CCP was upregulated and CAT was downregulated in the Δ*dnfA*, Δ*dnfB*, and Δ*dnfC* mutants, we were interested in the roles that CCP and CAT play in Dirammox. Thus, we created the Δ*CAT* and Δ*CCP* mutants. We performed ammonia conversion experiments with the WT, Δ*CAT*, and Δ*CCP* mutant strains by culturing them with 12.7 mM ^15^NH_4_^+^ as the sole nitrogen source at a 4:1 ratio of He/O_2_ (20% of O_2_) (Figure 3E,F). After 72 h of incubation, the levels of ^15^NH_4_^+^, ^15^NH_2_OH, ^15^NO_3_^–^, and ^15^N_2_ in all samples were determined. The results demonstrated that WT and the Δ*CAT* mutant generated similar amounts of ^15^N_2_ (17.8 ± 1.8% and 14.3 ± 0.2%, respectively) and ^15^N_2_O (4.8 ± 0.3% and 4.4 ± 0.2%, respectively). By contrast, the Δ*CCP* mutant converted more ^15^NH_4_^+^ into ^15^N_2_ (22.8 ± 1.9%), ^15^NO_2_^–^ (2.9 ± 0.1%), and ^15^N_2_O (12.1 ± 1.2%) compared with the WT and Δ*CAT* mutant. The WT and Δ*CAT* mutant strains did not accumulate ^15^NO_2_^–^. These data indicated that the silencing of the *CCP* gene, but not the CAT gene, resulted in ROS accumulation and enhanced ammonia oxidation to N_2_ and N_2_O.

### Dirammox is associated with intracellular ATP levels and affected by terminal electron transport

**Figure 4:**
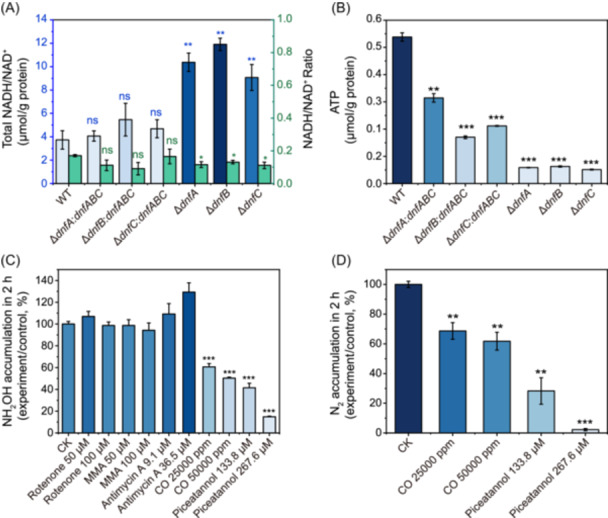
Association of the Dirammox pathway with the electron transport chain. (A, B) NADH/NAD^+^ ratio (A) and ATP level (B) of the JQ135 WT, Δ*dnfA*, Δ*dnfB*, Δ*dnfC*, Δ*dnfA*:*dnfABC*, Δ*dnfB*:*dnfABC*, and Δ*dnfC*:*dnfABC* strains cultured in HNM. The blue column chart represents the total NADH/NAD^+^ and the green column chart represents the NADH/NAD^+^ ratio in (A). (C) Level of NH_2_OH accumulation. The cells were treated with various inhibitors of the electron transport chain inhibitors for 2 h. (D) Level of N_2_ accumulation. Cells were treated with various concentrations of CO and piceatannol for 2 h. All statistically significant differences were determined using a Student *t*‐test. In (A) and (B), significance is based on comparisons to the WT group. In (C) and (D), the asterisk (*) means that the group is significantly lower than the CK group. ns, no significance; ***p < 0.001; **p < 0.01; *p < 0.05.

According to the proteomic profiling results, components of the terminal electron transport were significantly affected in the Δ*dnfA*, Δ*dnfB*, and Δ*dnfC* mutants, suggesting that the redox balance and energy metabolism in the Δ*dnfA*, Δ*dnfB*, and Δ*dnfC* mutants might differ from those of the WT. To assess this, we harvested bacterial cells when each strain reached an OD of approximately 0.9 and measured the total intracellular redox (NADH/NAD^+^) concentrations and ATP levels of the WT, Δ*dnfA*, Δ*dnfB*, and Δ*dnfC* mutant strains (Figure 4A,B). We found that the total intracellular redox concentrations of the mutants were much higher than those of the WT and complementary strains, even though the NADH/NAD^+^ ratios were similar in range (values ranged from 0.09 to 0.17). In addition, the ATP levels in the Δ*dnfA*, Δ*dnfB*, and Δ*dnfC* mutants were significantly lower than those of the WT and complementary strains. Taken together, these results demonstrated that the redox balance and the electron transport chain were disturbed in the Δ*dnfA*, Δ*dnfB*, and Δ*dnfC* mutants.

NADH molecules are the primary redox flux that fuels the terminal electron transport complexes for ATP generation. To obtain direct evidence demonstrating that Dirammox and ATP generation are associated, we treated cells utilizing Dirammox with specific inhibitors for each of the terminal electron transport complexes and examined the Dirammox activities. Inhibitors used in this study include rotenone, methylmalonate, antimycin A, CO, and piceatannol, which inhibit the activity of complexes I, II, III, IV, and V, respectively^30^, ^31^, ^32^. The results showed that rotenone, methylmalonate, and antimycin A were not effective on NH_2_OH accumulation. However, CO and piceatannol significantly reduced ^15^NH_2_OH accumulation and ^15^N_2_ production, and the inhibition was dosage‐dependent (Figure 4C,D). These results suggest that complexes IV and V of the terminal electron transport are directly related to the process of Dirammox.

## DISCUSSION

Several heterotrophic bacteria oxidize ammonia to NH_2_OH and then to NO_*X*_^–^ through a process called heterotrophic ammonia oxidation and heterotrophic nitrification. This microbial process has been known for decades^6^, ^8^ and has been widely utilized for the removal of nitrogen from wastewater^1^ and for understanding geobiological nitrogen recycling^33^. However, how and if heterotrophic bacteria benefit from this process are not clear. The oxidation of ammonia to NO_*X*_^–^ is an energy‐yielding reaction (∆*G* ^0^′ = −348.9 kJ mol^–1^, if NO_3_^–^ is the final product), and autotrophic ammonia‐oxidizing bacteria take advantage of this oxidation to generate ATP to support cellular metabolism and growth^34^. For example, the autotrophic ammonia oxidizer and nitrifier, *Nitrosomonas europaea*, yields 2 electrons per oxidizing 1 molecule of ammonia to NO_2_^–^ in Refs.^35^, ^36^, which subsequently fuels the terminal electron transport complex for ATP generation^37^, ^38^, ^39^. Efforts were made to confirm the existence of energy conservation during heterotrophic ammonia oxidation and nitrification^40^, ^41^, ^42^. Castignetti et al. observed that *A. faecalis* had a weak ability to generate protons from NH_2_OH oxidation^42^. In our study, we observed a lower NADH/NAD^+^ ratio and higher ATP levels in the WT ammonia‐oxidizing cells compared with Dirammox‐deficient cells (Δ*dnfA*, Δ*dnfB*, and Δ*dnfC* mutant strains) and demonstrated the association of intracellular redox flux and ATP turnover. We further observed that deletion of the Dirammox *dnf* genes resulted in a delayed growth effect (prolonged lag phase) when cells were transferred from LB to HNM broth. Thus, ammonia oxidation (Dirammox) plays an essential and physiological role in adaptation to environments by *A. faecalis*. Recently, Lv et al. proposed that Dirammox favored over cell growth of *A. ammonioxydans* HO‐1 by eliminating the toxicity of ammonia^22^. However, in this study, we observed that the growth of the mutants (Δ*dnfA*, Δ*dnfB*, and Δ*dnfC*) was not inhibited by increased ammonium concentration (Figure S5), suggesting that their result might require re‐evaluation. Intriguingly, we observed that ROS accumulated in the Δ*dnfA*, Δ*dnfB*, and Δ*dnfC* mutant strains, and the toxicity of ROS might also be responsible for the prolonged lag phases observed in these mutants.

**Figure 5:**
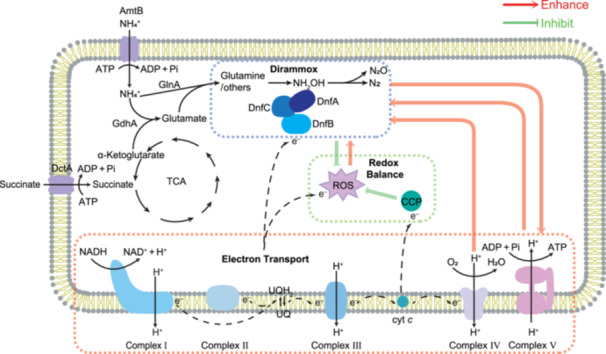
Physiological roles of Dirammox in *Alcaligenes*. Dirammox enhances electron transport and ATP production and controls intracellular redox balance. Dirammox is also influenced by terminal electron transport and intracellular ATP levels. CO and piceatannol can inhibit Dirammox activity by inhibiting complexes IV and V. In addition, our data indicated intracellular ROS advantages for Dirammox.

In both autotrophic and heterotrophic bacteria, ammonia oxidation proceeds stepwise via enzymatically catalyzed reactions. Ammonia is initially oxidized to hydroxylamine and then to NO, N_2_O, NO_2_^–^, and finally to NO_3_^–1^. The continuous oxidation of ammonia to NO_*x*_^–^ is called microbial nitrification and is catalyzed sequentially by AMO and HAO in autotrophic bacteria and archaea^3^, ^4^. Few studies have suggested, while most have taken for granted, that heterotrophic ammonia oxidation is catalyzed by similar AMO‐ and HAO‐like enzymes^33^, ^43^, ^44^, ^45^. The discovery of Dirammox revealed a different view on the biochemistry of heterotrophic ammonia oxidation because the enzymes DnfA, DnfB, and DnfC are not homologous to either AMO or HAO^16^. DnfA is a diiron‐oxygenase, and its involvement in ammonia oxidation was genetically demonstrated in *A. ammonioxydans* and *A. faecalis*. However, purified DnfA catalyzes the oxidation of hydroxylamine, but not ammonia, to N_2_ and trace amounts of NO_2_^–^ in Ref.^18^. We noticed that spontaneous chemical oxidation of hydroxylamine to N_2_ and N_2_O was proposed^20^, which might be a potential branch of Dirammox for N_2_ generation. However, we were concerned about the observed spontaneous chemical oxidation of hydroxylamine, since this apparent oxidation could be related to various ROS and ROS‐initiated oxidation. Generally, the sources of endogenous ROS are diverse, and bacteria growing aerobically generate ROS when O_2_ extracts electrons from reduced flavin, quinol, and transition‐metal functional groups^46^, ^47^. In this study, we observed that treatment with the ROS inhibitor BHT resulted in less NH_2_OH production, while treatment with the ROS inducer AAPH increased the production of NH_2_OH. We also observed that silencing of the *CCP* gene resulted in ROS accumulation and enhanced oxidation of ammonia to N_2_ and N_2_O. It is noteworthy that Δ*dnfA*, Δ*dnfB*, and Δ*dnfC* mutants had lower RNS levels compared with the WT and complemented mutants, suggesting that RNS may be involved in Dirammox in the WT. These findings and the physiological roles of ROS and RNS are summarized in Figure 5.

Importantly, the proteomic data from this study and transcriptomic results from previous study^22^ revealed that significant changes in the TCA cycle and oxidative phosphorylation were associated with intracellular and membrane‐associated electron transport chains. We confirmed that electron transport activity affected ammonia oxidation and Dirammox. Incubation with the electron transport inhibitors CO and piceatannol significantly decreased the production of NH_2_OH and N_2_, suggesting that complexes IV and V of the electron transport chain are involved in ammonia oxidation and Dirammox. CO inhibits the terminal respiratory proteins by forming a carbonyl complex with transition metals, including the heme‐prosthetic groups^48^, which are necessary for many biochemical processes. In *A. faecalis*, CO‐dependent inhibition might be partially neutralized due to the presence of the cytochrome *bd* complex, which is resistant to CO inhibition^32^. Piceatannol had the most powerful inhibitory effect on ammonia oxidation and Dirammox in this study. Piceatannol is a remarkable polyphenolic inhibitor of ATP synthesis^30^ and strongly interacts with the ATP synthase pocket^49^, ^50^. We also observed that piceatannol showed an inhibitory impact on both Dirammox and ATP synthase activities when incubated with ammonia as the sole nitrogen source. The process of Dirammox consists of the oxidation of NH_4_^+^ to NH_2_OH and NH_2_OH to N_2_. The oxidation of NH_4_^+^ to NH_2_OH is an ATP‐demanding process, and it has been recently disclosed the involvement of ammonia assimilation with glutamine synthetase^24^. The subsequent oxidation of NH_2_OH to N_2_ or NO_*x*_^–^ is mostly possible via an ATP‐generating process. Thus, Dirammox is a complicated process in terms of ATP metabolism, and further studies are needed.

In conclusion, our findings unveiled the association of Dirammox with intracellular redox balance and ATP metabolism and provided insights into the physiological role of Dirammox. We observed that endogenous ROS accumulated in Dirammox‐deficient cells and that silencing of the *CCP* gene in WT cells enhanced the N flux to Dirammox. Thus, we proposed that Dirammox would be upregulated by increasing ROS generation during rapid growth and that balancing ROS generation and energy metabolism would maintain rapid bacterial growth. As such, this may be a benefit for bacteria possessing the ability to utilize the Dirammox pathway (Figure 5).

## MATERIALS AND METHODS

### Chemicals and media

All reagents and chemicals utilized in experiments were commercially available. The HNM consisted of the following components per liter: 7.9 g of Na_2_HPO_4_·12H_2_O, 1.5 g of KH_2_PO_4_, 0.2 g of MgSO_4_·7H_2_O, 0.68 g of NH₄Cl, 4.72 g of sodium succinate hexahydrate, and 2 ml of trace element solution. The pH of the medium was in the range of 7.0 to 7.5. The trace element solution comprised the following per liter: 1 g of MnCl_2_·4H_2_O, 1.6 g of CuSO_4_·5H_2_O, 3.9 g of ZnSO_4_, 7 g of CaCl_2_·2H_2_O, 5.0 g of FeSO_4_·7H_2_O, 1.6 g of CoCl_2_·6H_2_O, and 57.1 g of EDTA‐2Na. LB broth consisted of the following components: tryptone (10.0 g/l), yeast extract (5.0 g/l), and NaCl (10.0 g/l) at pH 7.0.

### Bacterial strains, plasmids, growth conditions, and construction of mutants and complementary strains

All bacterial strains and plasmids used in this study are listed in Table S1. *E. coli* strains were grown at 37°C and *A. faecalis* JQ135 strains were grown at 30°C, both shaken at 160 rpm. Media were supplemented with kanamycin (Km, 50 μg/ml), gentamicin (Gm, 50 μg/ml), streptomycin (Str, 50 μg/ml), or chloramphenicol (Cm 50 μg/ml) as required. Complementary plasmids pBBR‐*dnfABC* were constructed by Gibson assembly based on pBBR1MCS‐5^51^, and the complemented strains were constructed through conjugation‐mediated transfer of the complementation plasmid into Δ*dnfA*, Δ*dnfB,* and Δ*dnfC* ^17^. Methods of base editing plasmids and Δ*CAT*/Δ*CCP* mutant construction are described in ref.^22^.

### Optical density growth curves

*A. faecalis* JQ135 strains were first activated overnight in LB broth until the stationary phase, and inoculated into HNM or LB at 0.5%(v/v) subsequently. 100‐well microtiter plates were filled with 200 μl of inoculated HNM and incubated in a Bioscreen C analyzer (Oy Growth Curves Ab Ltd.). At 30‐min intervals, the OD_600_ was measured. For each strain, 3 repetitions were performed. Growth curves were obtained by plotting the OD_600_ against the time.

### Microcalorimetric measurements

The respiration heat of strains was evaluated using a thermal activity monitor (TAM III; Järfälla), which is a multi‐channel microcalorimetric system. TAM III was used to record the heat flow rate of microbial growth precisely, and the thermal effect of the sample ampoule was adjusted to the electrical calibration. All samples were activated in LB broth and inoculated into HNM as described above. One milliliter of inoculated HNM was transferred into each ampoule and then the measurement was started. The usage of the microcalorimeter and the data analysis are described in ref.^52^.

### Data‐independent acquisition proteomics

#### Protein extraction, quality control, and enzymolysis

We performed harvesting when strains reached an OD_600_ ~ 0.9 by centrifugation (16,000 rpm for 30 min at 4°C). Four group samples were selected, and each group was represented by three biological replicates. Lysis buffer (1% SDS, 8 M urea, 1× Protease Inhibitor Cocktail [Roche Ltd.]) was added to the 12 samples, which were subsequently vibrated and milled for 40 s, three times. The samples were then lysed on ice for 30 min and centrifuged at 16,000 rpm for 30 min at 4°C. The supernatant was collected and transferred to a new tube and the concentration of protein was determined using Bicinchoninic acid (BCA) method by the BCA Protein Assay Kit (Thermo Scientific). The purity of the extracted proteins was verified by sodium dodecyl sulphate‐polyacrylamide gel electrophoresis on 12% gels, followed by Coomassie blue staining.

100 μg of protein was resuspended with triethylammonium bicarbonate buffer (TEAB) with a final concentration of 100 mM. The mixture was reduced with Tris (2‐carboxyethyl) phosphine with a final concentration of 10 mM at 37°C for 60 min and alkylated with iodoacetamide (IAM) with a final concentration of 40 mM at room temperature for 40 min in the dark. After centrifugation at 10,000*g* at 4°C for 20 min, the pellet was collected and then redissolved in 100 μl of 100 mM TEAB. Trypsin was added at a 1:50 trypsin‐to‐protein mass ratio and incubated at 37°C overnight. The peptide mixture was desalted by hydrophile lipophilic balance (HLB), quantified by the Pierce^TM^ Quantitative Colorimetric Peptide Assay (23275).

#### Liquid chromatography‐mass spectrometry analysis

Based on peptide quantification results, the peptides were analyzed by a VanquishNeo coupled with an Orbitrap Astral mass spectrometer (Thermo) at Majorbio Bio‐Pharm Technology Co., Ltd. Briefly, the ES906 column (150 μm × 15 cm; Thermo) was used with solvent A (water with 2% ACN and 0.1% formic acid) and solvent B (water with 80% ACN and 0.1% formic acid). The peptides were eluted using the 60 SPD gradient at a flow rate of 500 nl/min. Data‐independent acquisition (DIA) data were acquired using an Orbitrap Astral mass spectrometer operated in DIA mode. MS data were collected over an *m*/*z* range of 100 to 1700.

#### Data analysis for LC‐MS

Spectronaut software (Version 18) was used to search the DIA raw data. Six peptides per protein and 3 daughter ions per peptide were selected for quantitative analysis. The parameters are as follows: protein FDR ≤ 0.01, peptide FDR ≤ 0.01, peptide confidence ≥ 9 9%, and XIC width ≤ 75 ppm. The shared peptides and modified peptides were excluded, and the peak areas were calculated and summed to yield the quantitative results. Only the proteins with at least one unique peptide were used for protein identifications.

Bioinformatic analysis of proteomic data was performed with the Majorbio Cloud platform (https://cloud.majorbio.com). During the analysis, we applied a base‐2 logarithmic transformation to the raw data and conducted a normality test using the Shapiro–Wilk test and the D'Agostino *K* ^2^ test. The results demonstrated that the data followed a normal distribution. The *p*‐values and fold change (FC) for the proteins between the two groups were calculated using R package “*t*‐test.” The thresholds of fold change (>1.2 or <0.83) and *p* < 0.05 were used to identify DEPs. Functional annotation of all identified proteins was performed using the KEGG pathway (http://www.genome.jp/kegg/). DEPs were further used for KEGG enrichment analysis. Protein–protein interaction analysis was performed using the String v11.5.

### Detection of intracellular reactive oxygen species and reactive nitrogen level by flow cytometry

Total ROS levels were investigated by the fluorescent probe 2′,7′‐dichlorodihydro fluorescein diacetate (DCFH‐DA) (Aidisheng Biotechnology). RNS levels were detected using the bacterial reactive nitrogen detection fluorescent probe O52 (Beibo Biotechnology). O_2_^−^ levels were quantified using MitoROS™ 580 (AAT bioquest). H_2_O_2_ levels were monitored using the OxiVision™ Green peroxide sensor (AAT bioquest). OH^•^ levels were detected using the bacterial reactive nitrogen detection fluorescent probe O28 (Beibo Biotechnology). For DCFH‐DA, fluorescent probe O52, the OxiVision™ Green peroxide sensor, and fluorescent probe O28 were used; the fluorescence intensity was detected at an excitation wavelength of 488 nm and emission wavelength of 530 nm. For MitoROS™ 580, the fluorescence intensity was detected at an excitation wavelength of 561 nm and emission wavelength of 590 nm. Cells were harvested when strains reached an OD_600_ of 0.9 and then incubated with each probe at room temperature for 2 h; cells were washed again by 1× phosphate‐buffered saline and were then analyzed by a flow cytometer (BD LSRFortessa™). All tests were performed in triplicate.

### Analytical methods

When strains' growth reached an OD_600_ of 0.9, we harvested and lysed the cells to measure the concentrations of NAD^+^/NADH and ATP. NADH and NAD^+^ were detected using the NAD^+^/NADH Assay Kit with WST‐8 (Beyotime). ATP levels were detected using the ATP Luminescent Cell Viability Assay kit (Yeasen Biotechnology). The concentrations of ammonium nitrogen (NH_4_^+^‐N), hydroxylamine nitrogen (NH_2_OH‐N), nitrite nitrogen (NO_2_^−^‐N), and nitrate nitrogen (NO_3_^−^‐N) were determined following established standard protocols^16^, ^53^. Total nitrogen (TN) was determined using alkaline potassium persulfate digestion with ultraviolet spectrophotometry^54^. To determine the biomass nitrogen, bacterial cells for each of the samples were harvested by centrifugation (8000*g*, 10 min, 4°C) and resuspended in ultrapure water with the same volume as the original sample. The nitrogen content of this suspension was determined using the same method as that for TN determination and was calculated as the biomass nitrogen. Gaseous nitrogen products (^15^N_2_,^15^N_2_O) were analyzed using GC‐MS (model 7890 A/5975 C; Agilent). For nitrogen gas analysis, a CP‐Molsieve 5 A Plot column (25 m × 0.32 mm × 30 µm; Agilent) was used, while nitrous oxide was analyzed using a GS‐Carbon Plot column (30 m × 0.32 mm × 3.0 µm; Agilent). All tests were performed in triplicate.

## AUTHOR CONTRIBUTIONS

**Runhua Wang**: Investigation; methodology; software; supervision; validation; visualization; writing—original draft; writing—review and editing. **Xiaokang Wang**: Investigation; methodology; visualization. **Yue Zhao**: Investigation. **Xize Zhao**: Formal analysis; validation; visualization; writing—review and editing. **Tong Wu**: Data curation; formal analysis; validation; writing—review and editing. **Yulin Wang**: Data curation; formal analysis; writing—review and editing. **Ruofei Li**: Methodology. **Jun Yao**: Methodology; resources. **Chengying Jiang**: Writing—review and editing. **Ji‐Guo Qiu**: Validation; visualization; writing—review and editing. **De‐Feng Li**: Methodology; project administration; resources; supervision; writing—review and editing. **Shuang‐Jiang Liu**: Conceptualization; data curation; formal analysis; funding acquisition; project administration; supervision.

## ETHICS STATEMENT

No animals or humans were involved in this study.

## CONFLICT OF INTERESTS

The authors declare no conflict of interests.

## Supporting information

mLife SI.

## Data Availability

The raw data of proteomics generated in this study have been deposited in the iProX database under accession code IPX0010079000.
