# Supplementary material for: Heterotrophic ammonia oxidation by Alcaligenes balances ROS generation and terminal electron transport
Source: mLife. 2025 Sep 29;4(5):527–38. doi: 10.1002/mlf2.70035 (PMC12575085; doi:10.1002/mlf2.70035)
Supplement: Supplementary file 1 — mLife SI. [file MLF2-4-527-s001.docx]

**Heterotrophic ammonia oxidation by Alcaligenes balances ROS generation and terminal electron transport**

Run-Hua Wang^1,2^, Xiao-Kang Wang^1,2^, Yue Zhao^4^, Xi-Ze Zhao^1,2^, Yu-Lin Wang^3^, Ruo-Fei Li^4^, Jun Yao^4^, Cheng-Ying Jiang^1,2^, Ji-Guo Qiu^5*^, De-Feng Li^1,2*^, Shuang-Jiang Liu^1,2,3*^

**Email:**  Liusj@im.ac.cn

**This PDF file includes:**

Figures S1 to S4

Tables S1


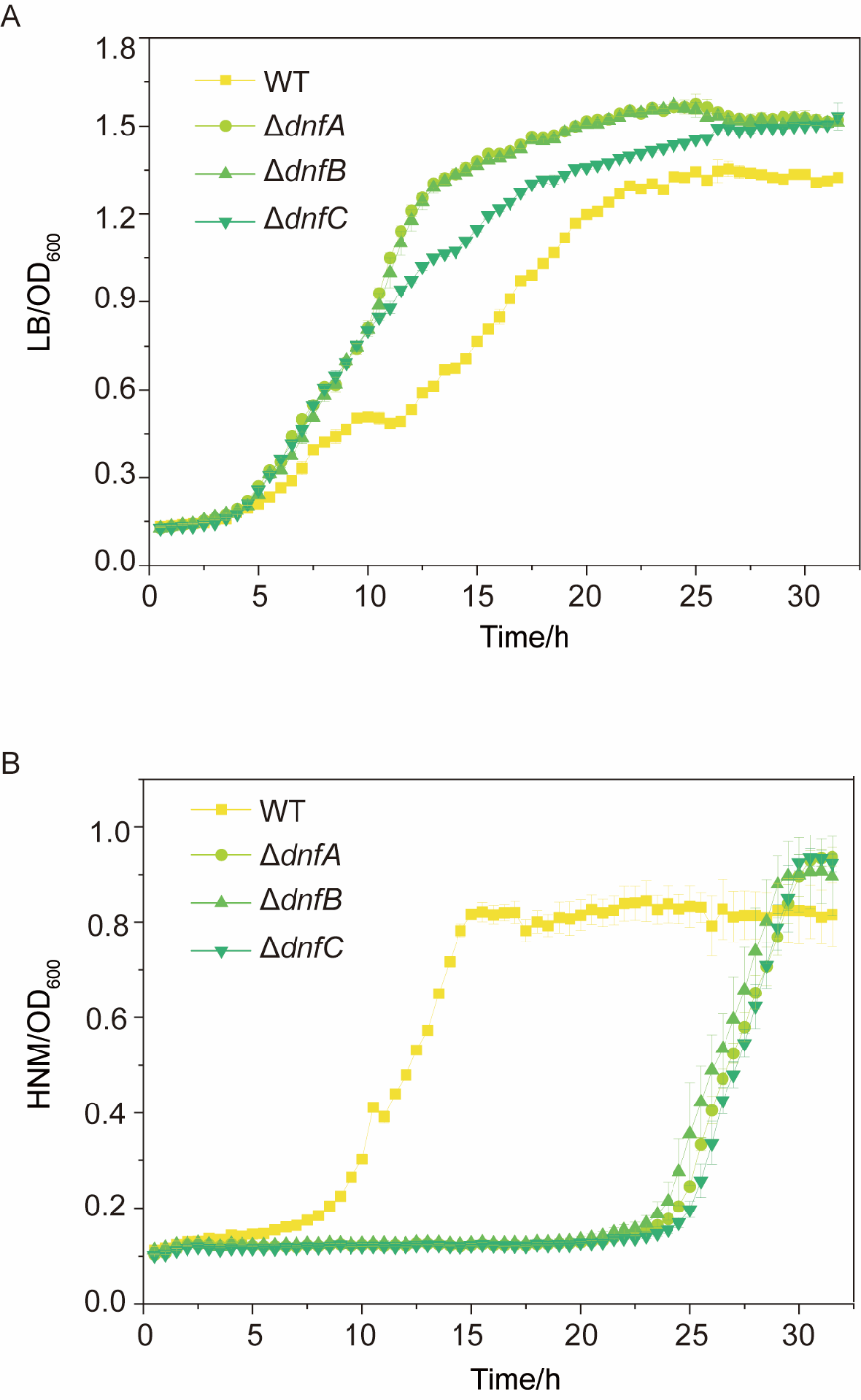


Fig S1. Growth curves of *A. faecalis* WT, Δ*dnfA,* Δ*dnfB* and Δ*dnfC* in LB (A) and HNM (B).


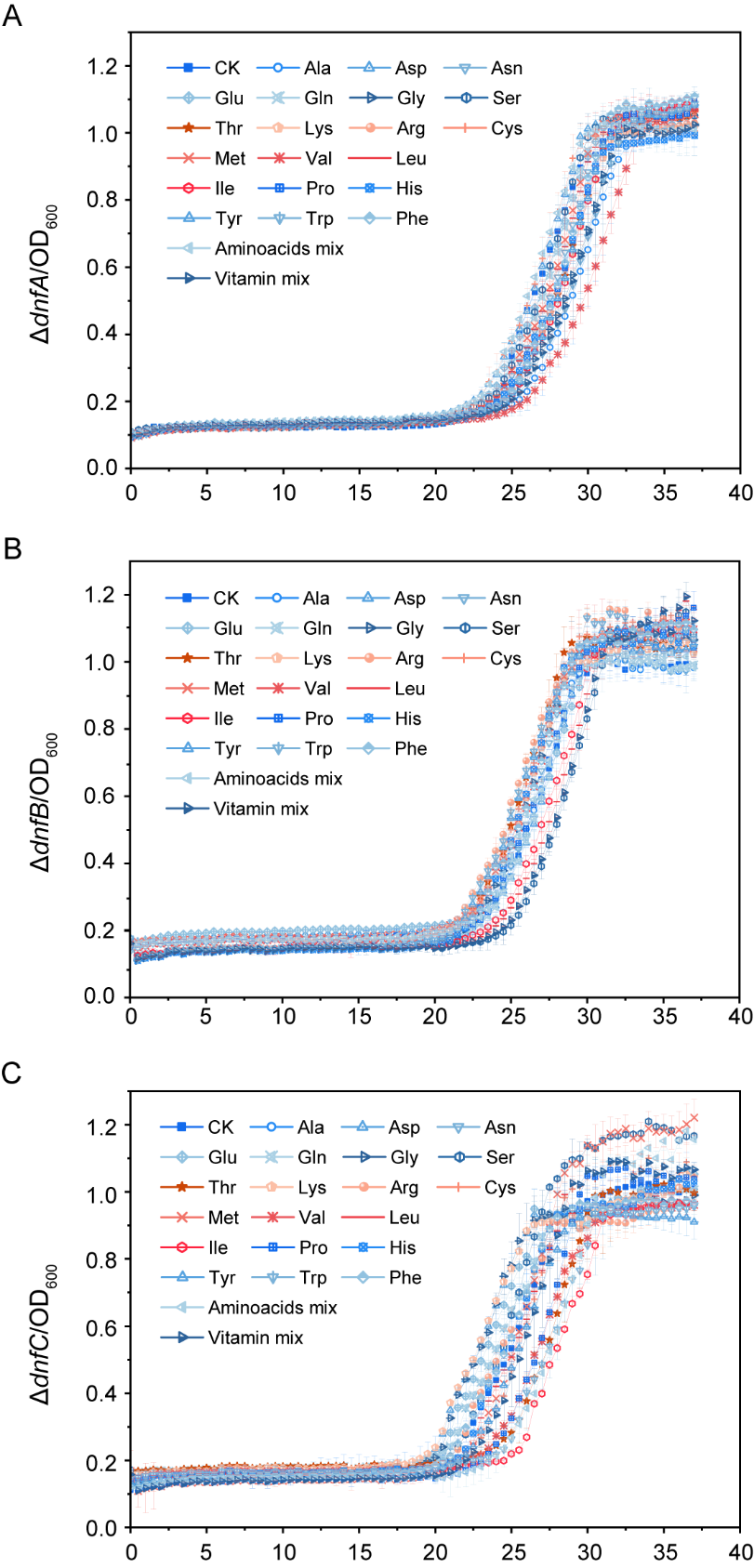


Figure S2. Growth curves of *A. faecalis* Δ*dnfA* (A)*,* Δ*dnfB* (B)and Δ*dnfC* (C) mutants in HNM supplement with 1 mM of 20 species of amino acids or vitamin mix.


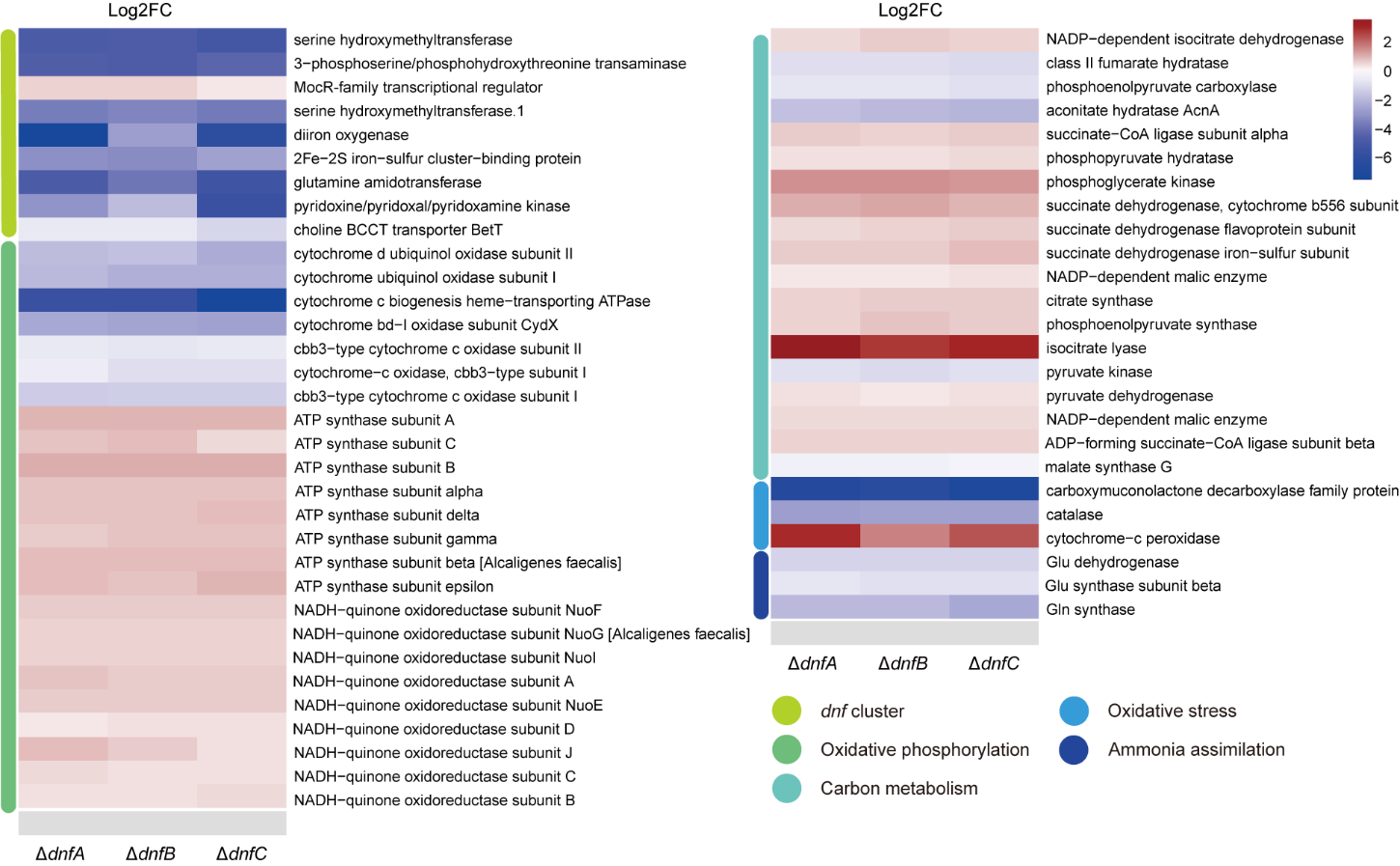
Figure S3. Heatmap of fold change of key metabolic proteins in Δ*dnfA,* Δ*dnfB,* Δ*dnfC* (comparing to WT).


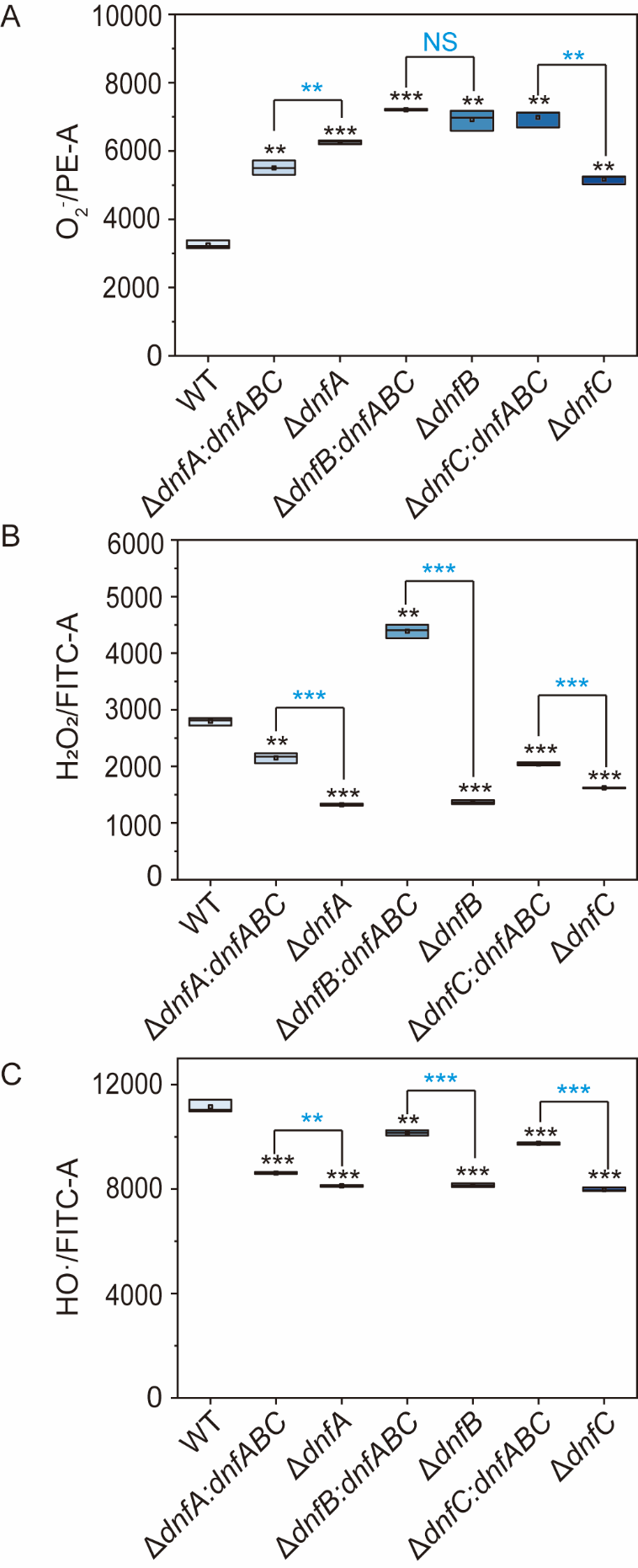


**Figure S4.** Detection of O_2_^‑^ (A), H_2_O_2_ (B) and OH• (C) in WT, Dirammox-deficient mutants (Δ*dnfA*, Δ*dnfB* and Δ*dnfC*) and complementary strains (Δ*dnfA:dnfABC*, Δ*dnfB:dnfABC* and Δ*dnfC:dnfABC*). Black asterisk represents statistically significant differences​ based on comparing to the WT group solely. Blue asterisk represents statistically significant differences​ between mutants and complementary strains. NS: no significant, *: p < 0.05, **: p < 0.01, ***: p < 0.001.


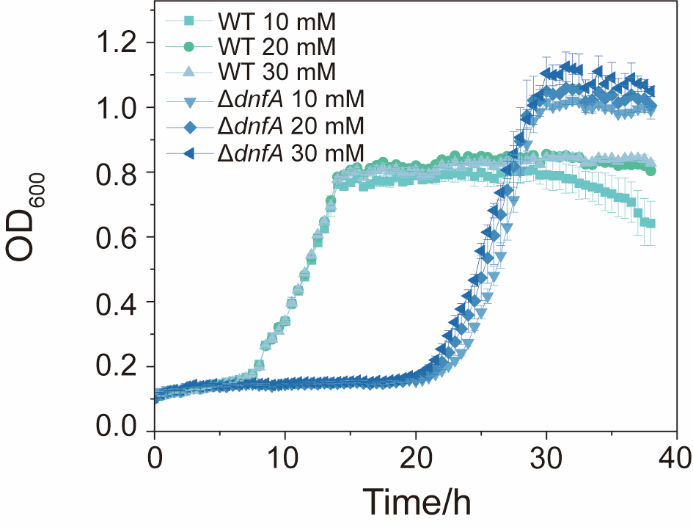


Figure S5. Growth curves of *A. faecalis* WT and Δ*dnfA* in HNM with 10, 20 and 30 mM NH_4_Cl.

**Table S1.** Strains, plasmids and primers used in this study

| Strains and Plasmids | Description | Source |
| --- | --- | --- |
| **Strains** |  |  |
| *A. faecalis* JQ135 | Str^­r^; wild type | 1 |
| JQ135 Δ*dnfA* | Str^­r^, Km^r^; *dnfA*-deletion mutant of JQ135; *dnfA*::Km^r^ | 2 |
| JQ135 Δ*dnfB* | Str^­r^, Km^r^; *dnfB*-deletion mutant of JQ135; *dnfB*::Km^r^ | 2 |
| JQ135 Δ*dnfC* | Str^­r^, Km^r^; *dnfC*-deletion mutant of JQ135; *dnfC*::Km^r^ | 2 |
| JQ135 Δ*CAT* | Str^­r^, catalase (*CAT*)-deletion mutant of JQ135 | This work |
| JQ135 Δ*CCP* | Str^­r^, cytochrome c peroxidase (*CCP*)-deletion mutant of JQ135 | This work |
| JQ135 Δ*dnfA:dnfABC* | Str^r^, Km^r^, Gm^r^; JQ135Δ*dnfA* containing pBBR-*dnfABC* | This work |
| JQ135 Δ*dnfB:dnfABC* | Str^r^, Km^r^, Gm^r^; JQ135Δ*dnfB* containing pBBR-*dnfABC* | This work |
| JQ135 Δ*dnfC:dnfABC* | Str^r^, Km^r^, Gm^r^; JQ135Δ*dnfC* containing pBBR-*dnfABC* | This work |
| DH5α | Cloning host | Lab stock |
| HB101(pRK2013) | Help strain for parental mating | Lab stock |
| **Plasmids** |  |  |
| pBBR1MCS-5 | Gm^r^ ; broad-host-range cloning plasmid | Lab stock |
| pBBR-*dnfABC* | Gm^r^ ; pBBR1MCS-5 harboring *dnfABC* gene | This work |
| pBE-chl | Cm^r^ ; base editor plasmid | 3 |
| pBE-*CAT* | Cm^r^ ; base editor plasmid for *CAT*-deletion | This work |
| pBE-*CCP* | Cm^r^ ; base editor plasmid for *CCP*-deletion | This work |
| **Primers** |  |  |
| BEC-CK-F | CCAGCAGTGCGGTAGTAAAGG | 3 |
| BEC-CK-R | ACAGCTCCAGCACATACAGGC | 3 |
| CAT-F | GTGGCAGTCAGTACAGCATGGCCA | This work |
| CAT-R | AAACTGGCC-ATGCTGTACTGACTG | This work |
| CCP-F | GTGGCTGCCAGCGATCACCGATGG | This work |
| CCP-R | AAACCCATCGGTGATCGCTGGCAG | This work |
| CAT test-F | GCCAGGCCAATAAAACGCC | This work |
| CAT test-R | AGATCGCGCTGCGAGC | This work |
| CCP test-F | AAGTTCCCTAAGCATCTCTGGAAAGAT | This work |
| CCP test-R | CAACTGCAAACGACCCATTATATCCAC | This work |

**SI References**

1 Qiu, J. et al. Identification and Characterization of a Novel pic Gene Cluster Responsible for Picolinic Acid Degradation in *Alcaligenes faecalis* JQ135. Journal of Bacteriology. 2019;201, DOI:10.1128/jb.00077-19.

2 Xu, S. Q. et al. Genetic Foundations of Direct Ammonia Oxidation (Dirammox) to N2 and MocR-Like Transcriptional Regulator DnfR in *Alcaligenes faecalis* Strain JQ135. Applied and Environmental Microbiology 2022;88:e02261-02221.

3 Lv, J.-L. et al. Direct ammonia oxidation (Dirammox) is favored over cell growth in *Alcaligenes ammonioxydans* HO-1 to deal with the toxicity of ammonium. Biotechnology and Bioengineering. 2024;121:980-990. DOI:https://doi.org/10.1002/bit.28623.
